# Titanium Particles Released from Dental Implants Under Fluoride Exposure Interact with Macrophages

**DOI:** 10.1590/0103-644020256187

**Published:** 2025-04-14

**Authors:** Waad Kheder, Soumya Sheela, A. R. Samsudin, Sausan Al Kawas, Nadia Khalifa, Ali Qabbani

**Affiliations:** 1 College of Dental Medicine, University of Sharjah,PO Box: 27272, Sharjah, UAE.; 2 Research Institute for Medical and Health Sciences, University of Sharjah, Sharjah P. O. Box. 27272, UAE

**Keywords:** Dentifrices, Acidic, Prosthesis, Elements, Solution, Inflammation

## Abstract

This study is designed to investigate the influence of fluoride and pH value on the release of titanium particles from Ti6Al4V dental implants with hydrophobic microrough surface produced by sandblasting and acid-etching techniques; and correlate particle size to their uptake by macrophages and expression of inflammatory cytokines. Fifteen dental implants were immersed in five test solutions with different fluoride concentrations and pH values. Three control implants were scanned using a scanning electron microscope and fifteen test implants were also scanned after their immersion in the test solutions. The immersion solutions were analyzed for titanium particles/ions size-range and amount. The uptake of titanium particles by macrophages and expression of Il-1 β and IL-8 following their exposure to titanium particles were investigated. Test solutions with high fluoride and acidity resulted in the release of micro-size titanium particles (4551.7 ± 114.5 nm and 2783 ± 101.13 nm); while those with low fluoride, neutral pH, and alkaline environment resulted in the release of nano-size titanium particles (431.2 ± 80.6 nm, 448.3 ± 112 nm, and 484.5 ± 85.3 nm respectively). There was an increase in the uptake of nanoparticles by macrophages without altering their membrane integrity. The increase in expression of IL-1β and IL-8 by M0 macrophages after exposure to titanium dioxide particles may facilitate our understanding of immune cell population-specific molecular events deriving the peri-implant inflammation in response to titanium particles. Fluoride and pH values influence the release of titanium particles from the implant's surface. The activated inflammatory mediators are key to imbalance in osteoblast-osteoclast activity and failure of implant osseointegration.

## Resumo

Este estudo foi projetado para investigar a influência do flúor e do valor de pH na liberação de partículas de titânio de implantes dentários de Ti6Al4V com superfície microrugosa hidrofóbica, produzida por técnicas de jateamento de areia e ataque ácido; e correlacionar o tamanho das partículas com sua captação por macrófagos e a expressão de citocinas inflamatórias. Quinze implantes dentários foram imersos em cinco soluções de teste com diferentes concentrações de flúor e valores de pH. Três implantes controle foram analisados por microscopia eletrônica de varredura, e quinze implantes de teste também foram analisados após a imersão nas soluções de teste. As soluções de imersão foram analisadas quanto ao tamanho e quantidade de partículas/íons de titânio. A captação de partículas de titânio pelos macrófagos e a expressão de IL-1β e IL-8 após a exposição às partículas de titânio foram investigadas. Soluções de teste com alto teor de flúor e acidez resultaram na liberação de partículas de titânio em microescala (4551,7 ± 114,5 nm e 2783 ± 101,13 nm); enquanto aquelas com baixo teor de flúor, pH neutro e ambiente alcalino resultaram na liberação de partículas de titânio em nanoescala (431,2 ± 80,6 nm, 448,3 ± 112 nm e 484,5 ± 85,3 nm, respectivamente). Houve um aumento na captação de nanopartículas pelos macrófagos sem alterar a integridade de sua membrana. O aumento na expressão de IL-1β e IL-8 pelos macrófagos M0 após a exposição às partículas de dióxido de titânio pode ajudar a compreender eventos moleculares específicos das células imunológicas que impulsionam a inflamação peri-implantar em resposta às partículas de titânio. O flúor e os valores de pH influenciam a liberação de partículas de titânio da superfície do implante. Os mediadores inflamatórios ativados são fundamentais para o desequilíbrio na atividade osteoblástica-osteoclástica e para a falha da osseointegração do implante.

**Figure:**
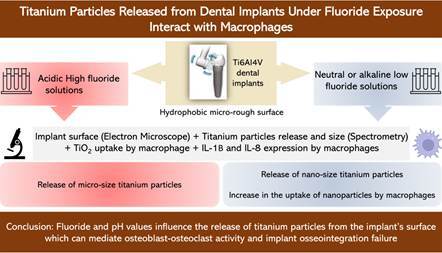

## Introduction

Titanium has proven successful as a base material in many implant-based medical devices such as hip prostheses and cardiac pacemakers where these devices are implanted deep in the body. Titanium and its alloys are also the material of choice in the manufacture of dental implants and their prosthetic parts to replace missing teeth. However, titanium-based dental prostheses can be exposed to the oral environment in cases where the implant is not completely covered by bone and/or adjacent soft tissue, and often need maintenance using fluoridated dentifrices. The successful clinical performance of titanium and its alloys is correlated to their biocompatibility and high corrosion resistance ^1^, and due to the 2-20 nm thick protective TiO_2_ film that is usually formed on the implant surface ^2^. This TiO_2_ layer has low reactivity with the surrounding milieu and can reduce ion release from implants to the biological environment ^3^. However, the exposure of titanium to the oral environment and corrosive substances such as fluoride may alter the titanium dioxide layer and expose the titanium leading to the degradation of dental implants and their prosthetic parts ^4^. Consequently, the degradation can significantly increase surface material loss leading to the failure of those implants or their supported prostheses ^5^.

Fluid and/or lubricated friction contacts commonly occur between the dental implant and its superstructure during loading and routine brushing of teeth with fluoride toothpaste. Furthermore, implant-supported prostheses reveal micro gaps at abutment-implant or abutment-prosthesis connections that can accumulate corrosive substances, such as fluoride, over a long duration, and end with corrosion of the titanium body ^7^. Oral fluoride application is one of the main methods for the prevention of dental caries and is present in many kinds of toothpaste and gels. The percentage of fluoride in these dentifrices often ranges from 0.1 to 2.0 wt. % and corrosion resistance of titanium-based prostheses may decline at these concentrations ^8^. The commercial dental rinses and gels containing 1000-10000 ppm fluoride with a pH between 3.5 and 7.0 are often used for caries-preventive prophylactic applications. However, the use of fluoride-containing rinses or gels might be harmful to titanium devices if the pH is below 7.0 and this may induce surface corrosion as a result nanoparticles (NPs) and/or microparticles (MP) can be released from the implants’ surface into the peri-implant area ^9^. Many studies have reported that these ions can diffuse into the bloodstream and lead to systemic cytotoxicity and mutagenic reactions ^10^. Besides, the release of metallic particles/ions at the bone-implant interface is also considered a risk factor for the development of peri-implant inflammation and failure of bone remodeling ^11^.

The research area of molecular and cellular interactions between tissues and dental implant interphase is expanding. Investigating the deformation of implant surface in combination with the application of cell biology is becoming an important tools to recognize how material properties are translated into different biological responses. The metallic ions that are released from the implant surface into the tissue may remain free in the peri-implant area. Otherwise, macrophages may contribute actively to the elimination of these foreign particles; and the presence of macrophages in failed implants has been associated with the formation of pathologically infected granulation tissue ^12^.

Immune cells such as lymphocytes, neutrophils, and macrophages, are present in the peri-implant gingival tissues ^13^. Titanium particles shed from dental implant surfaces have been identified as harmful foreign bodies surrounded by inflammatory cells in peri-implantitis tissues’ biopsies ^14^. Those particles are engulfed by macrophages that release pro-inflammatory cytokines such as IL-1β and IL-8 associated with osteoclast activation via the receptor activator of nuclear factor ĸB ligand (RANKL)/receptor activator of nuclear factor ĸB (RANK)/osteoprotegerin (OPG) signaling pathway; this ultimately leads to osteolysis and bone resorption ^15^. Most of the interactions between bone cells and immune system cells are modulated by cytokines, growth factors, and hormones ^16^. Cytokines have an undeniable role in the intercommunication of the immune cells in the inflammatory process leading to clinical manifestations ^17^. Imbalances in the levels of cytokine release may inhibit the resolution of inflammation and contribute to alveolar bone loss ^18^. Dental practitioners are unaware of implant leaching activity following exposure to the implant surface, use of acidic dental fluoride dentifrices, and action of microorganisms on titanium surfaces. Since these activities occur at the subclinical level, the practitioners are only aware when peri-implantitis develops. This study is designed to investigate the influence of fluoride and pH value on the release of titanium particles from dental implants, and correlate particle size to their uptake by macrophages and the expression of inflammatory cytokines.

## Materials and methods

### Dental Implants Selection

An immersion study was carried out to test the influence of fluoride concentration and pH value on the surface of titanium dental implants manufactured from titanium alloy (Ti6AL4V) with hydrophobic micro rough surface produced by sandblasting and acid-etching techniques for surface treatment, and to measure the size of the released titanium particles. A total of eighteen bone-level tapered-shaped dental implants with a dimension of 4.0×10 mm were used to compare the leaching of titanium particles/ions from implant surfaces following their immersion in five groups of solutions with different fluoride concentrations and pH values. Three implants were used as a control group and were scanned for surface and titanium element content using Scanning Electron Microscopy (SEM, VEGA3 XM - TESCAN) and energy dispersive X-ray spectroscopy (EDX, Oxford Instruments, Abingdon, United Kingdom). The remaining fifteen implants were scanned after immersion study, using scanning electron microscopy-energy dispersive x-ray (SEM-EDX).

### Test solutions preparation

Five different groups of test solutions were prepared for the immersion study; these solutions were chosen to mimic as possible most of the conditions inside the oral cavity that could affect the dental implant integrity. Each group comprise of three implants (n=3) immersed in three glass containers containing 20-ml of each solution.

- Test Solution I: 1.23 % Sodium Fluoride (NaF, Sigma-Aldrich) in phosphate-buffered saline (PBS) at a pH of 7.2.
- Test Solution II: Alkaline solution (prepared by adding 1 N NaOH to PBS and obtaining a pH of 9).
- Test Solution III: Acidic solution (prepared by adding conc. HCL to PBS and obtaining a pH of 3).
- Test Solution IV: 5 % NaF added to PBS at a pH of 7.2.
- Test Solution V: 1.23% acidulated phosphate fluoride (APF) gel at a pH of 3.5 (Henry Schein).

### Immersion Study

Titration towards alkaline and acidic conditions in test solutions II and III was obtained by adding NaOH and concentrated HCl (Sigma-Aldrich) to the respective PBS solutions. The temperature of the test solutions was maintained at room temperature. Three test implants were immersed in each group of the five test solutions, each implant placed in an individual glass container. The implants were subjected to continuous shaking in an electrical shaker for 7 days which represent cumulative exposure, approximating 5 years of the implants to thrice daily exposure with fluoride at different oral pH values during oral hygiene maintenance with mouth rinse or toothpastes. These 7 days of the simulation were calculated based on the routine individual oral hygiene measure of 2 min tooth brushing three times a day. Over a period of 5 years, this equals to a total of 10,080 min or 7 days ^6^.

### Implant surface analysis

On day 7 of the implant immersion period, all fifteen test implants were retrieved from the test solutions, rinsed with distilled water, and dried using an air syringe to remove the remnants of the test solution. SEM-EDX was used to investigate the changes in the implant surface as well as to quantify the post-immersion percentage of titanium element content on the surface of the implant in comparison to the control group. The SEM-EDX was operated at an acceleration voltage of 20 kV and 10 to 25 mm working distance. An SEM-EDX analysis was done in five different areas (SEM spectra) of the implant surface; three of them were on the first three threads of the crestal portion of the implant body, while two areas were in the valley between adjacent threads. A reference point was created by preparing a slot on the mount of the dental implant to ensure that the five selected areas are in the same vertical alignment. The difference in the percentages of titanium element content between control and test implants was calculated by adding the total values in the five spectra at the crestal part of the implant body and dividing by 5; the result represents the percentage difference of titanium element in the crestal portion. The same calculation was done for values in the apical part of the implant body. The differences in values between the test and control implants represent the percentage of titanium element loss after the immersion of the implant in the test solution.

### Titanium ions concentration analysis

Ten milliliters of each test solution were collected in 15 mL polypropylene tubes and used for Inductively Coupled Plasma Atomic Emission Spectroscopy (ICP-OES) analysis (Thermo Scientific™ iCAP™ 7400 ICP-OES Duo) to examine the concentrations of titanium ions released following implant immersion in test solutions. In this sample analysis, the ICP-OES was used together with an aqueous sample introduction port Teledyne CETAC™ ASX-280 autosampler. The analysis was performed in axial mode at a wavelength of 334.941 nm. The operating parameters are presented in Box 1. The concentrations of titanium ions were quantified in the solutions with a titanium standard (TraceCERT®, 1000 mg/L in 2% nitric acid) which covered a calibration curve ranging from 1-1000 ppb. The square relation coefficient (R2) is 0.9996 and the equation is y=0.4006x + 0.5066.

**Box 1:**
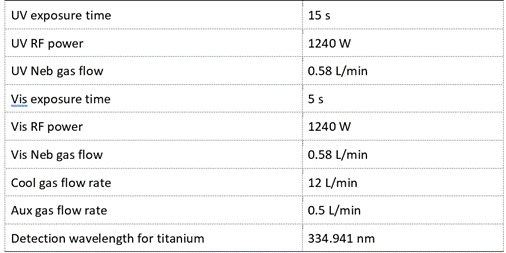
Parameters and operating conditions of ICP-OES employed for detecting titanium concentrations in test solutions

### Titanium particles size measurement

The size of titanium particles released in the respective test solutions during immersion study was measured at 25°C using a Malvern, Zetasizer Nano-ZS system (Malvern Instruments). Particles size was measured using DLS method where the scattered light was collected at 173°. The Z-Average value was considered as the mean diameter of particles.

### Titanium particles interaction with macrophages - titanium dioxide particles preparation

The interaction of different sizes of TiO_2_ particles with macrophages was evaluated by exposing them to different concentrations of TiO_2_ nanoparticles (TiO_2_-NPs) and microparticles (TiO_2_-MPs). Briefly, TiO_2_ NPs (size <100 nm, 634662) and TiO_2_ MPs (size <5 µm, 224227) in diameter were procured from Sigma (Sigma-Aldrich), and the TiO_2_ NPs and MPs were weighed and dispersed in Milli-Q water for stock preparation and were sonicated for 10 min with a probe sonicator (Qsonica sonicators). For cell culture studies, TiO_2_ particles were suspended in the complete Roswell Park Memorial Institute (RPMI) 1640 medium to a final concentration of 1 mg/ml (stock suspension), and 100 µg/ml was used as the treatment concentration. The hydrodynamic diameter, polydispersity index (PDI), and surface charge of TiO_2_ NPs and MPs were analyzed using a Malvern, Zetasizer Nano-ZS system before proceeding with the cell studies.

### Titanium particles interaction with macrophages - THP-1 monocyte-derived M0 macrophages cell line culture

THP-1 monocytes were obtained from CLS cell line services (CLS cell lines service GmbH, Eppelheim, Germany, catalog number: 300356, passage number 46) and maintained in RPMI-1640 medium supplemented with 10% fetal bovine serum (FBS), 100 U/mL penicillin and 100 µg/mL streptomycin. The cells were maintained in a humidified environment with 5% CO_2_ and 37°C. THP-1 monocyte cells were further converted into M0 macrophages using 100 ng/mL phorbol 12-myristate 13-acetate (PMA) (Sigma-Aldrich, USA) for 48 hours according to the protocol previously described by Chanput et al. 2014 ^19^. Following exposure to PMA, the cells were maintained in a PMA-free medium for 48-72 h. The flow cytometry analysis using CD14 was used to confirm the differentiation of THP-1 monocytes into M0 macrophages.

### Titanium particles interaction with macrophages - titanium particles uptake by macrophages

THP-1-derived macrophages were exposed to TiO_2_ NPs and MPs for 24 hours to analyze the interaction of the particle with the macrophages. The treated cells were washed with PBS and later ﬁxed in 2.5 % (v/v) glutaraldehyde prepared in PBS and were incubated for 1 hour at room temperature. The samples were further dehydrated using a gradient series of ethanol (from 50 to 100%) before sputter-coating with gold-palladium. SEM images of the particle interaction with the cells were acquired and the element composition of the particle in the cells was confirmed by EDX. The phase contrast microscopic images of the cells with particles were also acquired using an IX53 inverted microscope (Olympus, Shinjuku). 

### Titanium particles interaction with macrophages - IL-1B and IL-8 expression analysis by cytometric bead array

The alterations in the secretion of IL-1β and IL-8 by the THP-1 derived M0 macrophages after preferred treatments in the culture supernatants was quantified using the BD CBA Human Inflammatory Cytokines Kit (BD Biosciences, catalogue number: 551811). The analysis was done in a FACS Aria III flow cytometer using FCAP Array V3 software (BD Biosciences).

### Data analysis

The statistical significance of the test results was analyzed using one-way ANOVA followed by Tukey's post hoc test. Statistical analysis was performed using GraphPad Prism 9 software version 9.0.0 121 (GraphPad Software, LLC). A p-value of < 0.05 was considered statistically significant.

## Results

### Implant surface analysis

**Figure 1:**
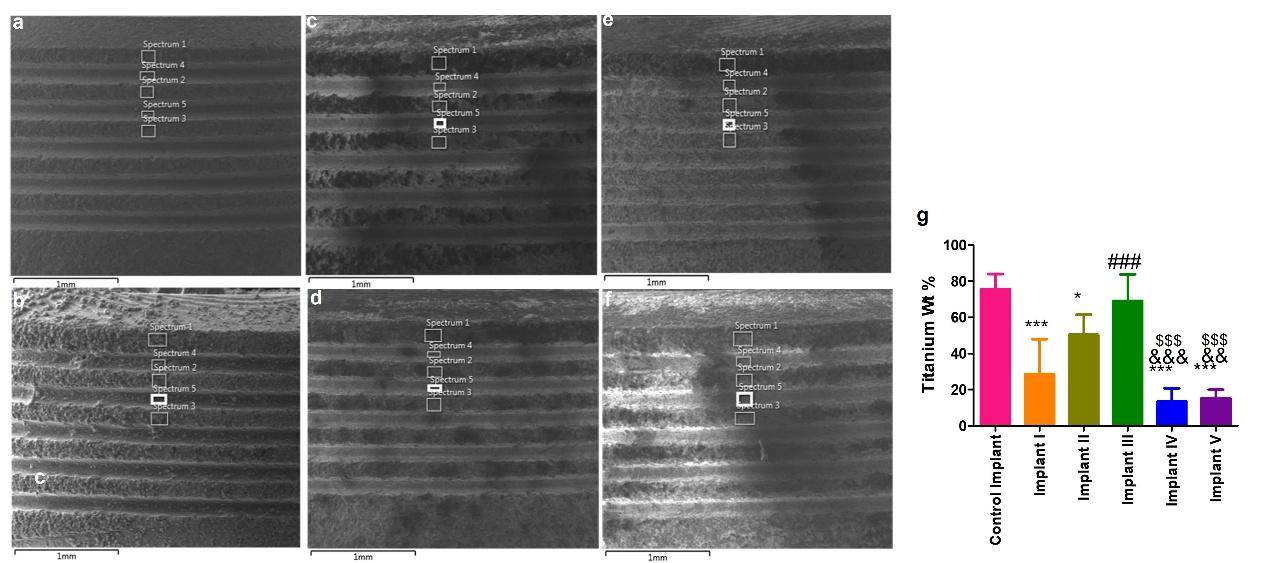
a-g: SEM images at crestal part of each implant surface following immersion study. The five selected spectra (1, 3, 5 in the crest of the threads, and 2, 4 in the valleys between threads) were at the same vertical alignment. (Figure 1a. Control implant, b. Implant I (in test solution I: 1.23 % NaF in PBS at pH 7.2), c. Implant II (in test solution II: Alkaline solution at pH 9), d. Implant III (in test solution III: Acidic solution at pH 3), e. Implant IV (in test solution IV: 5 % NaF in PBS at pH 7.2), f. Implant V (in test solution V:1.23% APF at pH 3.5). The average titanium percentage is represented as Mean ± SD in g. A statistically significant difference in the atomic weight. % of Titanium was found between the control implants group and the test implants group. *, **, and *** represent the significant difference between the control and test implants with p values <0.05, 0.001, and 0.0001 respectively. ### (p<0.0001) represents the analysis between implant I and implant III, &&& (p<0.0001) and && (p<0.001) represents the significance between implant II and implant IV and $$$ (p<0.0001) represents the difference between implant III with implant IV and V.

**Figure 2:**
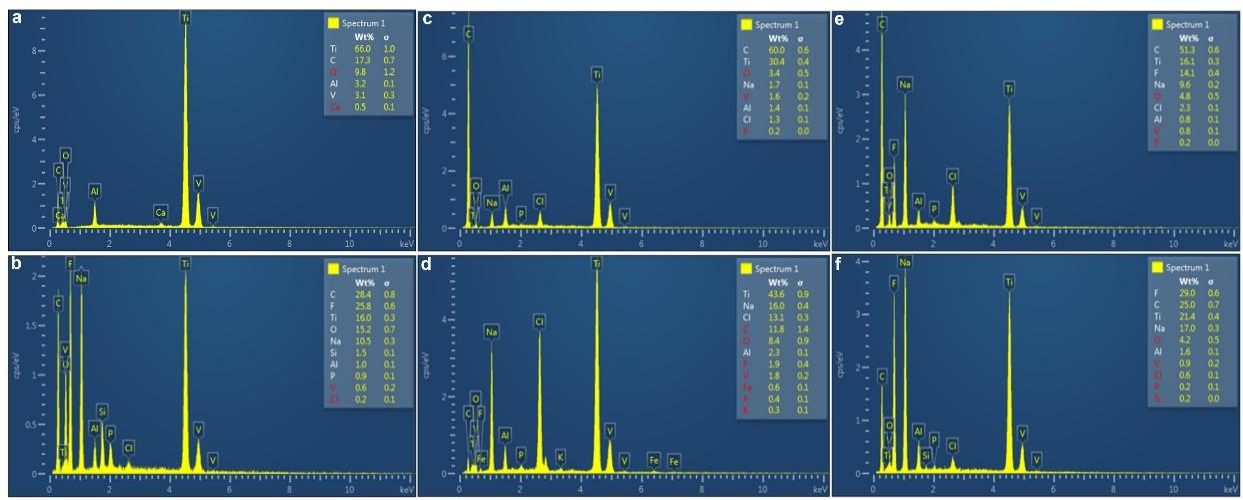
The post-immersion EDX analysis of all test implants (b, c, d, e, f). (Figure2a. Control implant, b. Implant I (in test solution I: 1.23 % NaF in PBS at pH 7.2), c. Implant II (in test solution II: Alkaline solution at pH 9), d. Implant III (in test solution III: Acidic solution at pH 3), e. Implant IV (in test solution IV: 5 % NaF in PBS at pH 7.2), f. Implant V (in test solution V:1.23% APF at pH 3.5).

The SEM-EDX analysis demonstrated differences in the percentages of titanium element content of each spectrum on the surface of the tested implants, after immersion into the test solutions (Figure1a-f and Figure 2 a-f)). All implants that were retrieved from test solutions showed a reduction in titanium element content in comparison to the control implant. The decrease in the percentage of titanium element content of the implants immersed in fluoride solutions (test solution I, IV, V) was higher compared to those immersed in individual alkaline and acidic solutions (test solution II, III) (Figure 1).

### Titanium ion concentration in test solutions

**Figure 3:**
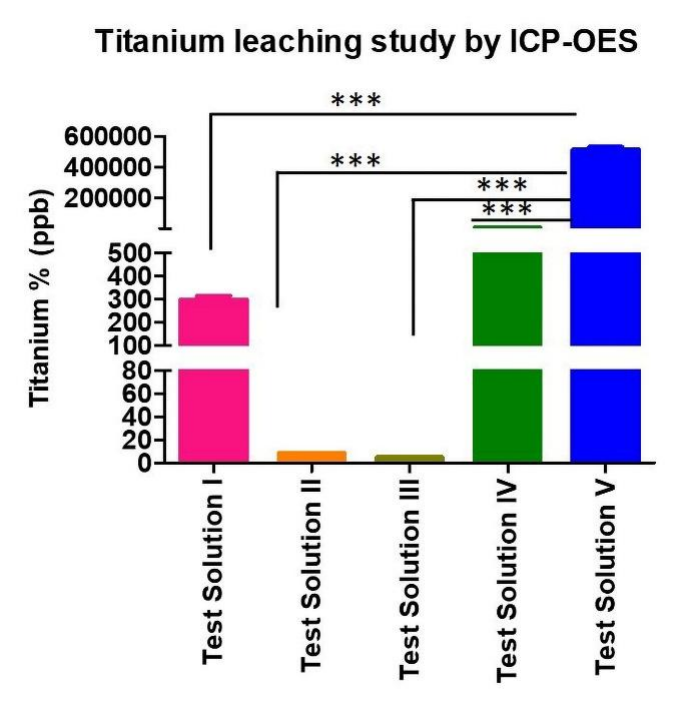
ICP-OES analysis showing titanium ions concentration following implant immersion in five different test solutions. Test solution I (1.23 % NaF in PBS at pH 7.2), test solution II (Alkaline solution at pH 9), test solution III (Acidic solution at pH 3), test solution IV (5 % NaF in PBS at pH 7.2), test solution V (1.23% APF at pH 3.5). The Data are represented as mean ± SD from three separate experiments. One-way ANOVA analysis showed a statistically significant difference in the titanium ion release between the test implants. *** represents p value <0.0001.

ICP-OES analysis confirmed the presence of titanium ions in all test solutions. The highest amounts of titanium ions were shown in the solutions containing fluoride (test solutions I, IV, and V) (Figure 3).

### Titanium particles in test solutions

Following the detection of titanium ions in test solutions using the ICP-OES analysis, the mean hydrodynamic diameter of titanium particles was measured. Test solutions IV and V demonstrated the presence of micro-size titanium particles (2783 ± 101.13 nm, 4551.7 ± 114.5 nm); while those in test solution I, II, and III confirmed the presence of nano-size titanium particles (448.3 ± 112 nm, 431.2 ± 80.6 nm, 484.5 ± 85.3 nm) (Table 1).

**Table 1:** Titanium particle size from the respective test solutions

Samples	Hydrodynamic Diameter (nm)
Test solution I	448.3 ± 112
Test solution II	431.2 ± 80.6
Test solution III	484.5 ± 85.3
Test solution IV	2783 ± 101.13
Test solution V	4551.7 ± 114.5

### Titanium particles characterization and uptake by macrophages

**Figure 4:**
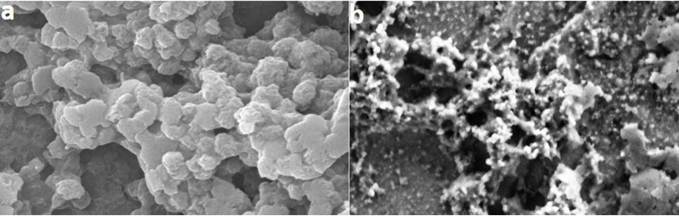
Representative SEM images of dispersed TiO_2_ (a) NPs and (b) MPs.

The size of TiO_2_ NPs examined from the SEM analysis was in the range of 150-300 nm (Figure 4a), whereas the TiO_2_ MPs showed an agglomerated morphology under SEM (Figure 4b). The DLS measurements of TiO_2_ NPs gave a mean hydrodynamic diameter of 322.83 ± 38.38 nm and the zeta potential values and PDI for TiO_2_ NPs were found to be -7.86 ± 0.28 mV and 0.36 ± 0.06. The hydrodynamic diameter, the zeta potential, and the polydispersity index (PDI) of TiO_2_ MPs were found to be 2723.33 ± 917.80 nm, -8.93 ± 0.37 mV, and 0.59 ± 0.07 respectively.

**Figure 5:**
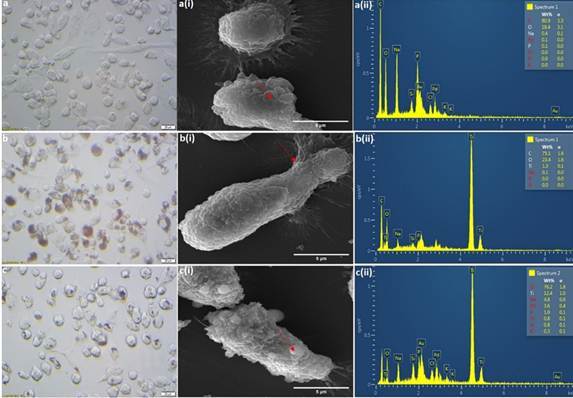
Phase contrast and SEM-EDX images showing the interaction of titanium particles with THP-1-derived M0 macrophages. A, b, and c are phase contrast microscopic images of control cells, cells treated with 100 µg/mL TiO_2_ NPs and100 µg/mL TiO_2_ MPs, respectively. The TiO_2_ particle uptake by M0 macrophages is clear from the images. a(i), b(i) and c(i) are SEM images of the control and activated M0 macrophages treated with 100 µg/mL TiO_2_ NPs and 100 µg/mL TiO_2_ MPs, respectively. The presence of titanium particles in contact with the cells was further elucidated from the EDX spectral mapping and clearly showed the presence of titanium for the NP (b(ii)) and MP (c(ii)) groups compared to the control (a(ii)). The red arrows refer to the areas selected for EDX analysis.

SEM studies demonstrated the uptake of TiO_2_ particles by macrophages, and the phase contrast microscopic images showed an increased uptake of NPs compared to MPs within the cytoplasm. The interaction with the TiO_2_ NPs and MPs did not alter the macrophage membrane integrity as is evident in the SEM images (Figure 5). The filopodia cellular extensions were clearly visible in the images. An elemental analysis by EDX confirmed the presence of titanium particles within the cells (Figure 5 b (ii) and c (ii)).

### IL-1B and IL-8 expression analysis by cytometric bead array

**Figure 6:**
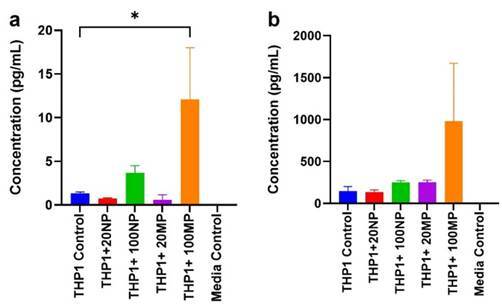
IL-1β and IL-8 protein expression by M0 macrophage after being treated for 24 h with 20 and 100 µg/mL TiO2 NPs and MPs using cytometric bead array. The expression of cytokines is represented in terms of picogram /milliliter to non-treated M0 macrophages. Data are represented as mean ± SEM from three separate experiments. * Represents p <0.05.

The IL-1β and IL-8 expression was investigated upon treatment with 20 and 100 µg/mL TiO_2_NPs and MPs for 24 h (Figure 6). An increase in the release of IL-1β (p<0.05) and IL-8 (not statistically significant) was noticed for the M0 macrophage group treated with 100 µg/mL TiO_2_ MPs compared to the untreated cells. Those cell groups treated with 100 µg/mL TiO_2_NPs also showed an increase in the IL-1β though not statistically significant in comparison to the control cells. However, between the NPs and MPs groups, 100 µg/mL TiO_2_ MPs showed a significantly higher expression for both the IL-1β and IL-8 expression.

## Discussion

Chronic inflammation and peri-implantitis a common causes of long-term implant failure. A host of reasons have been attributed to this condition including poor surgical techniques, overloading of implant-supported prosthesis, imbalance occlusion, and titanium wear particles. Although particle disease has long been associated with osteolysis and implant failure in orthopedic surgery, the impact of titanium particles-induced aseptic peri-implant cytotoxicity has not much been discussed in dental implant practice. However, the microbial biofilm consists of an important etiological factor of peri-implant pathology analogous to periodontal diseases, and this could be the septic cause of peri-implant infection. Furthermore, recent observations have shown that fluoride and a high-acidity environment play a role in modifying the titanium dioxide protective layer on the surface of the implant leading to the release of titanium particles ^20^.

In the present study design, the duration of implant immersion in test solutions simulated the condition in the oral cavity where the teeth are usually exposed to fluoride three times/d during teeth maintenance using mouth rinse and fluoride toothpaste; and taking into consideration the changes in pH of oral cavity while consuming different food products. However, a variety of immersion frequencies (e.g., twice per day) and concentrations of NaF mouth rinse (0.05% to 0.5%), and (1.23%) APF gel have also been used in previous studies ^21^. The cumulative 5-year period was taken following studies that showed a dental implant failure rate of 12% over that period, while a common acceptable prognosis for dental prostheses is in the range of 10 year’s lifetime ^22^.

This study revealed that there was a release of titanium particles which is an indication of the loss in corrosion resistance of dental implants surface following immersion in solutions of different fluoride types and concentrations at pH ranging from 3 to 9. The results of the ICP-OES analysis showed the presence of titanium ions in all immersion solutions, the highest being in a fluoridated solution of low pH (test solution V). This phenomenon reflects the role of fluoride concentration and pH value in the destruction of the TiO_2_-based passive film on implant surfaces due to the attack of fluoride ions which leads first to corrosion and then to the release of titanium particles. Reclaru and Meyer 1998, identified the formation of a soluble titanium-fluoride complex (TiF6 complex) that leads to a decrease in the resistance of the protective oxide layer to corrosion. Besides, the release of titanium particles is also related to the nature of the immersion solutions, when highly acidic solutions are used, the fluoride ion combines with the H+ ion to form hydrofluoric acid, which consequently enhances the release of titanium particles in an acidic environment. Schiff et al. 2002 also demonstrated that hydrofluoric acid is reactive to titanium surfaces even when the NaF concentration is low, and titanium alloy is not able to withstand exposure to a NaF solution exceeding a concentration of 5,000 ppm.

It is known that increased fluoride concentration leads to increased porosity and decreased thickness of the oxide layer, which reduces its corrosion protection ^23^. Toumelin-Chemla et al. 1996, found that the presence of fluoride ions in a neutral (pH 6.75) fluoride solution of 10,000 ppm does not delay the formation of a protective layer, although the protective oxide film has been degraded in the acidic and fluoridated environment ^24^. Our finding suggests the importance of acidic pH in the release of titanium particles from dental implants as the percentage of titanium ions in the acidic test solution at pH 3 (test solution III) was less than that for test solutions containing fluoride with an acidic pH of 3.5 (test solution V). This is to the findings by Veys-Renaux et al. 2016, who demonstrated that the ingestion of acidic beverages can decrease oral pH, and may also acidify the pH of saliva, contributing to the corrosion of dental implants and the release of titanium particles ^25^.

This study also demonstrated the effects of fluoride in the release of particles from dental implants even when the pH is neutral. Our results showed that there is release of titanium ions with 1.23% and 5% NaF solution at neutral pH 7.2. This is in agreement with Bhattarai et al. 2008, who found that the degradation of dental implants and their associated prostheses depends on reactive factors present in the oral environment such as fluoride ^26^. Pröbster et al. 1992, demonstrated that tarnishing of titanium did not occur when the pH of the solution was equal to or higher than neutral. Furthermore, in prophylactic gels or solutions containing 400 to 9000 ppm fluoride, corrosion of titanium was often easily identified because the pH of these gels and solutions was usually lower than neutral ^27^.

The present study showed that APF at a concentration of 1.23% and pH 3.5 (test solution V) demonstrated an increased leaching of titanium ions compared to NaF at a concentration of 1.23% or 5% at pH 7.2 (test solution I, IV). This was very vivid in our ICP-OES analysis, whereby the concentration of titanium particles was higher in 1.23% APF solution than in 1.23% NaF solution. This confirmed the impact of acid in enhancing the release of titanium particles from implant surfaces. Our study introduced a new approach to evaluate and confirm the loss of titanium elements on the surface of the implant following immersion in different test solutions. The results of this approach, using the SEM-EDX system, showed consistency with the findings of ICP-OES when analyzing for the loss of titanium element on the surface of the implants and detecting these ions in the test solutions. Consequently, in the SEM-EDX analysis, the titanium element content percentages in the implant immersed in test solution V was less than that in implants immersed in test solutions II and III, while the ICP-OES results showed higher titanium ions concentration in test solution V than that in test solution II and III.

The size of the released particles does exert a biological impact on the response of macrophages phagocytosis ^28^. Accordingly, we studied the relation between the size of titanium particles (found in the test solutions) and fluoride concentration and pH in the test solutions. In test solutions IV and V, the particles were in micro size, while in test solutions I, II, and III, the particles were in the nano range (Table 1). This finding can be interpreted as that the exposure of titanium to high fluoride concentration (5%) and/or too low pH environment increased the release of micro size particles (test solutions IV&V), (Table 1); while exposure to lower fluoride concentration (1.23%) and/or to high pH environment led to the release of nanoparticles size (test solution I, II, III), (Table 1). The range of particle size selected for the uptake study (322.83 ± 38.38 nm for NPs and 2723.33 ± 917.80 nm for MPs) was within the range of the particle size released in this immersion study. Our study demonstrated that the macrophages uptake of TiO_2_ NPs was more than MPs, and the interaction with the TiO_2_ NPs and MPs did not alter the macrophage membrane integrity, as is evident in the SEM images.

Instead of using titanium plates and discs as proposed by Chen et al. in a previous study ^28^, our study utilized actual titanium dental implants, which can simulate the clinical scenario. Although the dental implant is not exposed to the oral environment and should be well embedded in the alveolar bone, around 0.9 - 1.2 mm of it will be unprotected from the oral environment due to the marginal bone loss after one year of loading the implant, in addition to the inevitable 0.2-mm bone loss per year after one-year loading. Besides, in case of peri-implant bone loss, the implant’s surface will be exposed to the variables in the oral environment such as fluoride and acidity. In this study, the friction contacts between three surfaces, namely the implant, the surface of the glass container, and the immersion solution during implant shaking could have attributed to the generation of kinetic energy which converts to thermal energy and subsequently contributes to the release of titanium particles from dental implant. This phenomenon agrees with findings by Landolt (2006) who advocated that contact geometry and type (sliding, rolling) determine the degradation rate of dental implants and their prosthetic infrastructures ^2^.

In the clinical scenario, the whole immune cells in the peri-implant gingival tissues are usually exposed to titanium wear particles. However, our in vitro experiments targeted only the macrophage, which is the main cell included in the response to foreign body reaction, to investigate the reflection of interaction between macrophages and titanium particles. This experiment was crucial to confirm the impact of titanium particles in the expression of inflammatory cytokines such as IL-1β and chemokines such as IL-8. IL-1β is related to the direct differentiation of pre-osteoclast into osteoclast that is influencing the failure of dental implant osseointegration. On the other hand, IL-8 attracts neutrophils and activated macrophages to the periprosthetic site and impairs bone homeostasis which ultimately ends up in bone loss over time.

The test solutions that contain titanium particles released from implant surfaces cannot be used in the treatment of cells in vitro. So, to simulate the clinical scenario, different concentrations of nano and micro-sized TiO_2_ particles were used in the present study in the treatment of THP-1 monocyte-derived M0 macrophages, to reflect the interaction between titanium particles and macrophages. The results confirmed, at both gene and protein levels, that TiO_2_ particles stimulate macrophages to secret IL-1β, and this agrees with the finding of a study by Pettersson et al who showed that IL-1β is a pro-inflammatory mediator expressed by macrophages. The immunological reaction to titanium particles is complicated and depends on the interaction between exposed immune cells and the expression of different cytokines which induce accordingly the polarization of pro-inflammatory macrophages ^29^. 

The increase in the expression of pro-inflammatory cytokines by macrophages suggests the causal relationship between titanium particle release and failure of dental implants. This was in correlation with previous findings showing that IL-1α and IL-1β affect the activation of osteoclast and correlate to the downregulation of type 1 collagen; thus, it is related to the resorption of bone during peri-implantitis ^17^. Besides, in our study, there was an increase in the expression of IL-8 chemokine following the treatment of M0 macrophages with TiO_2_ particles. IL-8 is a well-established chemokine marker of peri-implant osteolysis ^30^. Though it is also expressed by human osteoblasts with implant debris around the peri-implant area, the primary cells responsible for the production of IL-8 are human macrophages ^31^.

Based on the present experimental findings, patients who wear titanium-based implant and prostheses should be informed about the negative effect of high fluoride concentration associated with acidic substances. However, this effect will depend on the frequency and concentration of fluoride release. Therefore, dentists should review the clinical history of the patients to determine the local and systemic factors that may induce low pH in the oral cavity.

It was impossible to simulate the oral cavity environment 100% using in vitro studies. Furthermore, the authors couldn’t use titanium particles that were collected from the experimental solutions as this option is not valid due to: 1) The quantity of the collected particles was not enough to be used in the treatment of the cells in vitro; 2) It was impossible to separate the nano from the micro titanium particles in the collected solution. Besides, the study model didn't take into consideration the continuous removal of fluoride and titanium ions by saliva and swallowing. This natural process could significantly reduce the concentration of corrosive substances in contact with the implants between brushings times, lowering the potential for titanium particle release. Besides, it was impossible to apply the salivary buffering effects in this study model.

The strength of this study was using a direct, cost effective and simple method to investigate the effects of fluoride concentration and pH values on the release of titanium particles from dental implant. Also, measuring the size of the released particles and further analyzed the interaction of the particle with macrophages.

## Conclusion

Under the current experimental design of this study, all tested implants showed the release of both nano and micro-sized titanium particles under different test conditions. Both acidity and fluoride concentration influenced the release of titanium particles and ions. However, the presence of fluoride in low acidity showed a synergistic effect on titanium release from the dental implants’ surfaces. Moreover, the macrophage uptake of TiO_2_ NPs was higher than MPs without altering the cell's membrane’s integrity. This was a reason for a high increase in the level of IL-1β and IL-8 expression by macrophages after exposure to different concentrations of titanium dioxide particles. This finding would facilitate our understanding of immune cell population-specific molecular events driving peri-implant inflammatory response to titanium particles.
